# Sexual and reproductive health of women living with HIV in Muslim-majority countries: a systematic mixed studies review

**DOI:** 10.1186/s12914-020-00225-z

**Published:** 2020-03-11

**Authors:** Dyah Juliastuti, Judith Dean, Lisa Fitzgerald

**Affiliations:** 1 School of Public Health, Faculty of Medicine, University of Queensland, Brisbane, Australia; 2 Ichsan Medical Centre Bintaro Health College, South Tangerang, Indonesia; 3 School of Public Health room 111, Faculty of Medicine, The University of Queensland, 288 Herston, Road, QLD 4006, Brisbane, Australia; 4 School of Public Health room 025, Faculty of Medicine, The University of Queensland, 288 Herston, Road, QLD 4006, Brisbane, Australia; 5 School of Public Health room 021, Faculty of Medicine, The University of Queensland, 288 Herston, Road, QLD 4006, Brisbane, Australia

**Keywords:** WLHIV, Muslim-majority countries, Sexual and reproductive health, Mixed methods

## Abstract

**Background:**

The number of women living with the human immunodeficiency virus (WLHIV) in Muslim-majority countries has increased significantly in the last decade. These women are often marginalized and face insecure sexual and reproductive health (SRH) needs and rights. However, little is known about the multi-faceted factors influencing these women’s fertility, contraceptive, and perinatal decisions and sexual life. This systematic mixed studies review aimed to synthesize the empirical evidence on social, cultural, and structural factors influencing the SRH of WLHIV in Muslim-majority countries.

**Methods:**

This review provides a synthesis of quantitative, qualitative and mixed-method research findings searched from PubMed, EMBASE, Scopus, CINAHL and Cochrane databases. We screened 3452 SRH studies involving WLHIV. The studies, published in English between 2008 and 2017, were from 20 Muslim-majority countries with increased numbers of WLHIV. The quality of eligible studies was appraised using a mixed-methods appraisal tool (MMAT) version 2011. Findings were thematically analysed by a hybrid deductive-inductive approach. Two independent reviewers were involved in the study selection, data extraction, quality appraisal, and data synthesis.

**Results:**

We included 13 SRH-related studies involving 1748 WLHIV in eight Muslim-majority countries. Most of these studies explored fertility desire and sexual health, while only a small proportion related to contraceptive use and the perinatal-care experience. We identified that WLHIV faced neglect of their SRH rights. These rights were predominantly affected by the socio-cultural, religious and health-services context of the women’s lives, which directed them to unsafe sex practices and stressful perinatal experiences.

**Conclusions:**

This study points to the need for SRH laws, policies, and interventions which stop WLHIV experiencing SRH discrimination violence and achieving their SRH rights.

## Background

In 2018, nearly half of the people living with human immunodeficiency virus (HIV) globally were estimated to be women of reproductive age [1]. UNAIDS estimated that the total number of women living with HIV (WLHIV) increased from 15.7 million in 2008 to 18.8 million in 2018 [1, 2]. Increasing access and uptake of antiretroviral therapy (ART) among WLHIV during this period have contributed to a significant decrease in AIDS-related deaths among WLHIV and in the incidence of mother-to-child-transmission [3]. However, women and girls across the globe remain among the most vulnerable to contracting HIV and other adverse sexual and reproductive health (SRH) outcomes [4]. In the last decade, although numerous countries have reported declining rates of new HIV transmission [5, 6], in many regions with predominantly Muslim populations, such as the Middle East and North Africa, the rates of new HIV cases have continuously increased, particularly among women [5, 7–9]. Heterosexual sexual contact remains the predominant mode of HIV transmission among women and men in most countries of these regions [7, 9, 10]. However, there is also significant incidence from male-to-male sexual contact and drug use among women and/or their sexual partners [7, 9, 11, 12].

Similar to the general population of women, promoting and achieving optimal SRH is essential for WLHIV, for not only their well-being but also the well-being of their partner/s and children [13]. WLHIV should have the ability to make decisions about their own reproduction and sex life, including the right to freely choose whether or not to have children, how many to have and when to have them and to have access to integrated health services promoting care and attention to SRH and prevention of HIV and other sexually transmissible infections (STI) [14, 15]. Protecting WLHIV’s SRH rights is fundamental for the women’s well-being [15], and a key strategy for reducing maternal-to-child transmission [16, 17] and HIV-related mortality and morbidity [18].

A strong connection between politics and religion restricts the SRH rights of women in the Muslim world [19, 20]. Gender-related power differences set within traditional Muslim socio-cultural-religious structures, combined with laws and policies that fail to protect the autonomy and sexual rights of women, restrict women from seeking SRH prevention, treatment, and care services [21–24]. For WLHIV, such vulnerabilities and discrimination are compounded. Strong social and cultural norms and beliefs, combined with prevailing HIV related stigma among community and health care workers that attributes HIV to immoral sexual activity [7, 8] creates reluctance among women to disclose their HIV status to family and barriers to accessing HIV treatment and care and other much needed SRH services [25–27]. Women who engage in commercial sex work, extramarital sexual activities, and HIV risk-related behaviours considered culturally ‘taboo’ and immoral are most vulnerable to experiencing barriers to HIV and SRH services and care [9, 27]. Nevertheless, there a dearth of evidence and contextualised understanding of the complexity of factors influencing the SRH and well-being of WLHIV in Muslim-majority countries.

This review aims to explore and synthesize the empirical evidence on social, cultural, and structural factors influencing the SRH decisions of WLHIV in Muslim-majority countries. It seeks to enrich understanding of SRH constraints of this marginalized population and guide the development of appropriate health policies and interventions.

## Methods

A systematic review was conducted to synthesize qualitative and quantitative data on the complex social, cultural, and structural factors influencing SRH among WLHIV in Muslim-majority countries. For the purposes of this paper Muslim-majority country is defined as a country in which more than 50% of the total population are people who follow or practice Islam [28]. A systematic mixed studies review was chosen to provide comprehensive findings and better understanding of the complex SRH issues of WLHIV [29–31].

### Search strategy

**Fig. 1:**
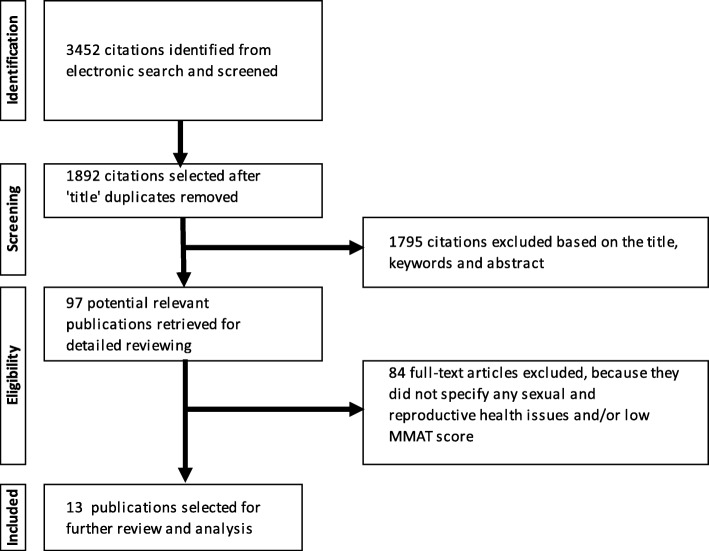
Systematic research

A systematic search of a range of medical, public health, and social science research databases including PubMed, EMBASE, Scopus, and CINAHL was conducted from September 2017 to February 2018. The search was limited to papers published in a ten-year period (2008–17) to obtain the latest overview of evidence. The following keywords were used to identify publications reporting findings of studies exploring the SRH of WLHIV in Muslim-majority countries [28]: (wom* OR female) AND (HIV OR AIDS) AND name of the country (such as “Iran”). The Cochrane Database of Systematic Review also employed the MESH Terms: (“Women OR woman OR female,” “HIV OR AIDS” and “Muslim OR Islam*”) to find similar systematic reviews. Selected HIV, social science, and medical journals, such as *AIDS* and *The Lancet,* were checked for references cited in key retrieved papers to identify additional papers. Details of the search strategies are presented in Fig. 1.

### Inclusion criteria and study selection

After identifying potential papers through a systematic search and removing duplicates, the titles, keywords, and abstracts were screened and assessed using the following inclusion criteria: (1) the study focused on SRH issues, such as fertility desire, contraceptive use, perinatal care experiences, and sexual life, of WLHIV with or without their partner or men living with HIV as the sample; (2) the study method was either qualitative, quantitative, or mixed; (3) the study was conducted in a Muslim-majority country where the estimated number of WLHIV had tended to increase from 2008 to 2017 [28, 32](See Table 1); and, (4) the paper was written in English. The results of this process were imported into Endnote. The full-text articles were re-screened and assessed independently by the three authors to obtain the final selection.

**Table 1:** Proportion of Muslim population, HIV prevalence, and number of WLHIV in the Muslim countries included in identifying potential articles. Adapted from Pew Research Centre (2011) and UNAIDS (2018)

No.	Country	Muslim population (%)*	HIV prevalence (%) in adult population (15–49 years)**		Estimated number of WLHIV**	
			2008	2017	2008	2017
1	Afghanistan	99.8	…	…	…	…
2	Algeria	98.2	0.13	< 0.1	2300	6100
3	Bangladesh	90.4	0.18	< 0.1	2600	4400
4	Chad	55.7	1.7	1.3	53,000	62,000
5	Egypt	94.7	< 0.1	< 0.1	1800	4800
6	Indonesia	88.1	0.3	0.4	120,000	220,000
7	Iran	99.7	< 0.1	0.1	11,000	15,000
8	Kazakhstan	56.4	< 0.1	0.2	2100	8000
9	Kyrgyzstan	88.8	0.1	0.2	1000	2500
10	Lebanon	59.7	< 0.1	< 0.1	< 500	< 500
11	Malaysia	61.4	0.5	0.4	14,000	17,000
12	Morocco	99.9	< 0.1	< 0.1	5900	7800
13	Niger	98.3	0.5	0.3	15,000	16,000
14	Pakistan	96.4	< 0.1	0.1	12,000	43,000
15	South Sudan	71.4	2.9	2.4	81,000	98,000
16	Tajikistan	99.0	0.2	0.3	2100	3800
17	Tunisia	99.8	< 0.1	< 0.1	< 500	< 1000
18	Turkey	98.6	…	...	…	…
19	Uzbekistan	96.5	0.2	0.3	9700	17,000
20	Yemen	99.0	…	…	…	…

* Pew Research Center [28]

**UNAIDS [32]

### Methodological quality appraisal

A formal quality assessment of the studies included into this review was conducted using the McGill Mixed Methods Appraisal Tools (MMAT) version 2011 [33]. This tool has been tested for its reliability and efficiency [34, 35] and has been utilized in several related publications [36–38]. Results of the quality assessment are presented in Table 2.

**Table 2:** Research summary of the included publications

***Author/year of study***	***Country***	***Study Type & Sample***	***Aim of Study***	***Main Themes***					Quality Score**
				***Fertility Desire***	***Contraceptive Use***	***Perinatal Experience***	***Safer Sex***	***Sexual Life***	
Adam et al., 2016 [39]	Sudan	Quantitative (descriptive study) / 26 WLHIV*	To investigate the maternal characteristics, pregnancy outcomes and estimate of maternal to child transmission of HIV among HIV infected women.	x		x			25%
Anwar et al., 2010 [40]	Bangladesh	Sequential mixed method / 15 PLHIV* (Qualitative); 38 WLHIV & 76 MLHIV^1^ (Quantitative)	To explore sexual life of people with HIV/AIDS in Bangladesh	x	x		x	x	75%
Behboodi-Moghadam et al., 2015 [41]	Iran	Qualitative (content analysis) / 15 WLHIV	To explore fertility intentions and experiences of infected women with HIV	x		x		x	75%
Behboodi-Moghadam et al., 2016 [42]	Iran	Qualitative (content analysis) / 12 pregnant WLHIV	To explore the experience of pregnancy among Iranian women with HIV	x		x			75%
Davis et al., 2017 [43]	Kazakhstan	Quantitative (cross-sectional study) / 242 WLHIV	To determine the extent which HIV-positive women are trading sex and to identify risk factors that may drive participation in sex trading among this population					x	75%
El Fane et al., 2011 [44]	Morocco	Quantitative (descriptive study)/72 WLHIV & 62 MLHIV	To identify sexual disorders affecting PLWHA and to determine factors influencing their sexuality				x	x	50%
Jiwatram-Negron et al., 2017 [45]	Kazakhstan	Quantitative (cross-sectional survey) / 249 WLHIV	To explore whether similar or different patterns of risk and protective factors emerge					x	75%
Kaplan et al., 2016 [46]	Lebanon	Qualitative (constant comparative) / 10 WLHIV	To develop a framework that facilitates understanding of the process by which women created meaning in their lives during and after learning of their sero-positivity.	x					75%
Karaosmanoglu et al., 2011 [47]	Turkey	Quantitative (descriptive study)/27 WLHIV & 109 MLHIV	To determine the epidemiologic and clinical features of patient with HIV infections and AIDS followed during a 3.5-year period.				x		50%
Mohammadi et al., 2015 [48]	Iran	Qualitative (content analysis)/10 WLHIV	To explore the lived experience of domestic violence in Iranian HIV-infected women					x	75%
Nedjat et al., 2015 [49]	Iran	Mixed method descriptive sudy / 25 WLHIV & 20 MLHIV (Qualitative); 160 WLHIV & 240 MLHIV	To evaluate the sexual and reproductive health needs of PLHIV in Tehran	x	x	x	x	x	100%
Rahmalia et al., 2015 [50]	Indonesia	Quantitative (prospective cohort) / 881 WLHIV & 1781 MLHIV	To determine the relative proportion of female patients in an HIV cohort and characterized their probable transmission route and reproductive profile.		x		x		75%
Saeieh et al., 2016 [51]	Iran	Qualitative (content analysis) / 18 WLHIV	To explore experience s of HIV positive women about contraceptive use	x	x		x	x	75%

*WLHIV Women living with HIV, *PLHIV* People living with HIV, *MLHIV* Men living with HIV

**Mixed Methods Appraisal Tool version 2011 [30]. The score is 25% when QUAL = 1 or QUAN = 1 or MM = 0; it is 50% when QUAL = 2 or QUAN = 2 or MM = 1; it is 75% when QUAL = 3 or QUAN = 3 or MM = 2; and it is 100% when QUAL = 4 and QUAN = 4 and MM = 3 (QUAL being the score of the qualitative component; QUAN the score of the quantitative component; and MM the score of the mixed methods component

### Descriptive data extraction

An Excel matrix was compiled from details of the selected publications: author name and year of study, country, study type and sample characteristics, aim of study, key findings, the knowledge gaps of the papers, and MMAT-quality score. These details were then compared and synthesized (See Table 2).

### Data synthesis

A convergent qualitative synthesis was adopted to structure the review in which the quantitative, qualitative, and mixed methods data were transformed into qualitative themes, concepts, and patterns using a qualitative thematic synthesis [30]. Initially, we listed predefined themes in which we assigned the data from the eligible studies. After all the data were compared and contrasted, similar results were grouped together into particular themes, and coded to formulate new themes [30, 31] (See Table 2). This thematic analysis was conducted using a hybrid deductive-inductive approach [30]. To ensure consistency and reliability of data synthesis, data analysis was conducted by the first author and independently reviewed by the remaining two authors. Disparities were resolved by discussion and consensus among the three authors.

## Results

### Characteristic of studies

A total of 3452 records were identified from the database search. Figure 1 shows the literature search and elimination process conducted as per the MMAT guidelines; 97 potential full-text articles were read in detail by the reviewers, and 13 were identified for inclusion. The 13 articles, published between 2010 and 2017, reported findings from studies conducted in eight of the 20 eligible Muslim-majority countries, including Iran, Turkey, Lebanon, Sudan, Morocco, Bangladesh, Kazakhstan, and Indonesia. Tables 2 and 3 present summaries of the basic details of the 13 studies. The 13 papers included in this review consisted of four qualitative (30.8%), six quantitative non-randomized and descriptive (46.1%), and three mixed-methods studies (23.1%). Sample sizes ranged from 10 to 881 WLHIV who were interviewed or completed an individual questionnaire in a hospital (69.2%) or in a community-support group context. The authors of five papers (38.5%) reported on studies conducted in Iran [41, 42, 48, 49, 51], and five studies included an exploration of the SRH of men living with HIV [40, 44, 47, 48, 50].

**Table 3:** General characteristics of the included publications

Description	Specification	Number of Study
Study Type	Qualitative	4
	Quantitative	6
	Mixed Method	3
Country of origin	Bangladesh	1
	Iran	5
	Indonesia	1
	Kazakhstan	2
	Lebanon	1
	Morocco	1
	Sudan	1
	Turkey	1
Study setting	Hospital	9
	Community-supporting group	2
	Mixed setting	2

### Key themes

Five key themes were identified from the data synthesis: (1) fertility desire; (2) c*ontraceptive use and decision-making*; (3) perinatal experiences and outcomes; (4) condoms and safer sex practice; and (5) sexual satisfaction and life after HIV. The summary of factors related with these themes can be seen in Table 4.

**Table 4:** Synthesis findings: factors influencing the sexual and reproductive health among WLHIV in Muslim-majority countries

Fertility Desire	Contraceptive Use	Perinatal Experiences	Safer Sex Practice	Sexual Life
• Individual satisfaction and hope for better future by having child • Having living child • Having HIV-infected child • Fear of transmitting the infection to the baby • Worried of child’s well-being and future • Uncertainty about their-own health and well-being • Pressure from husband/ family to conceive • Social and cultural belief about having child after married • Stigma and discrimination experience of previous pregnancy and delivery • Pressure from health care providers for not having (more) pregnancy	• Women’s fertility desire • Fear of contraceptive’s side effects • Religious belief constrictions • Partner’s preference of contraception • Less access to the methods • Lack of methods’ availability • Limited information and understanding about varied contraceptive methods • Pressure from health providers to use male condom only • Legal abortion services	• Trusting the God will • Complying HIV- treatment • Health care provider discriminated behaviour • Health care providers showed less respect and no confidentially • Access to safe abortion services • Lack of PMTCT information • Non-economical formulae feeding	• Fertility desire • Unknown HIV-status of sexual partner • Status disclosure to sexual partner • Inconvenience in using and procuring condoms • Fear of partner violence if persuading condom use • Lack of agreement with partners related to condom use • Patriarchal belief and engendered norms • No access to female condom • Limited information about condom use and its efficacy	• Feeling guilty of having pre-marital sexual activities • ART uptake • Sexual violence • Denial of HIV status • Not wanting to be pregnant • Fear of infecting sero-discordant partner • Worried to be divorced and lack of social and economic support from partner • Mental illness • Drug use

### Fertility desire

For WLHIV, fertility desire, defined as a desire to have children in the future, was complex and varied by parity and family pressure [40–42, 46, 49, 51]. Some Iranian [41, 49], Lebanese [46], and Bangladeshi [40] WLHIV expressed a strong desire to have children. The desire of respondents and their partners to have children influenced the low rates of consistent condom use reported in a number of the studies [40, 49]. For women who did not have a child prior to their diagnosis, the decision to have children was a way of achieving satisfaction and stability in their life [49]. For some Iranian WLHIV, pregnancy and motherhood were considered a primary reason for continuing their life and proof that they were ‘complete women’, with many considering motherhood an essential element in stabilizing their relationship with their spouse [42, 46]. In a Lebanon study [46], WLHIV described how the chance to experience motherhood and have children generated hope for the future and meaning in their lives.

Conversely, some other WLHIV in Iran expressed no fertility desire [41, 42, 49, 51], describing reasons such as having a living child, having children living with HIV, concern about transmitting HIV to their baby, fear of their children being harmed if their HIV diagnosis was disclosed, uncertainty about the future and their health, and experiences of stigma and discrimination from healthcare providers during their previous pregnancy as key contributing factors [41, 42, 46, 49]. Low fertility desire was also influenced by perceived and enacted pressure from healthcare providers to not become pregnant [41] and to terminate pregnancies [49]. However, a strong underlying cultural expectation to reproduce led some women to report being forced by their partner and family to become pregnant and experiences of being threatened with divorce and/or violation by their partner when they expressed their desire to have no children or stop having more children [41]. Lebanese WLHIV who had children prior to their HIV diagnosis expressed relief when they were not forced to reproduce [46].

### Contraceptive use and decision-making

Contraceptive choices of WLHIV were similar across studies conducted in Iran, Indonesia, and Bangladesh [40, 49–51], with condoms emerging as the most frequently used method of contraception over hormonal or other methods of contraception, though the prevalence of contraceptive use varied. A study by Nedjat et al. [49] found most respondents (80.3% of 160 WLHIV) reported using only one contraceptive method, either condoms (31.3%), or hormonal or permanent contraceptives (23%); while 15.8% reported using dual methods, combining condom use with another modern method. An Indonesian study of 881 WLHIV by Rahmalia and colleagues [50] found 43.9% used condoms and 22.5% used non-condom modern contraceptives. Among those in monogamous relationships, there was a low rate of contraceptive use, particularly among married couples [50]. Rahmalia et al. attributed these low rates to embedded sociocultural norms and religious values that place significant importance on having many children. In a study in Bangladesh by Anwar et al. [40], 67% WLHIV respondents, who were predominantly married, were using condoms inconsistently. Inconsistent condom use was reported to be associated with desire for parenthood [40, 49].

WLHIV’s contraceptive decision-making was influenced by a range of social and cultural beliefs [40, 49–51]. Partner’s contraceptive preferences and fertility desire, relationship power differentials, religious beliefs and norms restricting contraceptive use, and women’s strong desire to have children were factors reportedly limiting the use of modern contraceptives [40, 49, 51]. Respondents in the two studies in Iran by Nedjat et al. [49] and Saeieh et al. [51] were reluctant to use hormonal or permanent contraception methods due to fear of side effects and the misperception that non-condom modern contraceptives caused infertility and increased their risk of acquiring STIs and other genital infections.

The shortage of reproductive health services where WLHIV felt that their SRH needs were understood also influenced their contraceptive decision-making [49, 51]. Limited access to contraceptive information and poor availability of options resulted in poor understanding and usage of the different methods suitable for WLHIV [49, 51]. For example, healthcare providers’ suggestion to use male condoms as the primary contraceptive interfered with the uptake of hormonal and permanent contraceptives [49, 51]. In one study reviewed, the majority (86.5%) of WLHIV reported that they had never heard of emergency contraception [49]. Limited knowledge of emergency contraception and other contraceptive choices has been linked with experiences of unintended pregnancy post-HIV diagnosis [49, 51]. Only the studies in Iran examined access to legal abortion services for WLHIV if they had an unintended pregnancy [49, 51]. The availability of safe abortion services for WLHIV in Iran has reportedly led many to consider abortion as an acceptable means of family planning instead of using modern contraceptive methods [49, 51].

### Perinatal experiences and outcomes

Studies from Sudan and Iran [39, 41, 42, 49] suggested that WLHIV experienced high-risk pregnancy and stressful perinatal events post-HIV diagnosis. The Sudan study by Adam and colleagues [39] found that respondents had higher rates of anaemia during pregnancy, spontaneous preterm births, and neonatal deaths than the control group of HIV-negative women. Pregnant Iranian WLHIV commonly experienced psychological distress concerning the impact of pregnancy on their health and that of their baby [41, 42, 49]. Some Iranian WLHIV described how distress associated with previous antenatal, delivery and postpartum care experiences influenced their fertility intention [41]. They also reported fear and anxiety associated with the need to disclose their HIV status to their family and others and being referred to referral care services [41, 42, 49].

Inadequate social and structural support produced perinatal anxiety for WLHIV [41, 42, 49]. Iranian respondents described distress associated with experiences of stigmatization, discrimination, and being labelled as a sex worker by health providers. Many attributed these experiences to their HIV status and decision to have a pregnancy post-diagnosis [41, 42, 49]. Anxiety and perinatal distress were also triggered by the lack of information provided to WLHIV about how to prevent mother-to-child transmission [49]. In Nedjat and colleagues’ study [49], less than 32% of respondents recognized the modes of perinatal HIV transmission and how to prevent it. During the postnatal period, the compulsory formula feeding for the baby enforced by healthcare providers raised financial concerns due to the cost of purchasing the formula [41, 49]. The limited number of referral hospitals providing specialist perinatal services for WLHIV was reported to cause difficulties for WLHIV in accessing comprehensive and adequate services for themselves and their HIV-positive children [41]. Complying with antiretroviral therapy regimens was reported to prevent perinatal distress, however, some women discussed allaying their anxiety about the baby’s health and their own health by trusting in God’s will [42]. Others described how terminating their pregnancy using legal abortion services alleviated the stressors associated with an unintended pregnancy [49, 51].

### Condoms and safer sex practice

Studies conducted in Iran, Turkey, Bangladesh, Morocco, and Indonesia involving mostly WLHIV who were married and in monogamous relationships highlighted limited awareness of safer sex practices and inconsistent condom during sexual contact, despite condoms being the most common contraceptive used by WLHIV and promoted by the health care providers [40, 44, 47, 49–51]. Several social, cultural, and structural factors determined the inability of WLHIV to use condoms consistently [40, 49–51]. Iranian and Bangladeshi WLHIV affirmed their fear of partner violence if they advocated for condom use [51]. Lack of agreement with partners related to condom use [49] and partner’s parenthood desire [40, 49] were barriers to using condoms consistently. The study in Bangladesh [40] suggested that men’s dominant position within relationships deterred WLHIV’s ability to use condoms effectively, negotiate condom use, and refuse condomless sex and other unsafe sexual practices. Women’s fear to talk about sexuality and disclose their HIV status to their sexual partner, their inability to procure condoms, and their partner’s refusal to use a condom also impeded the consistent use of condoms [40, 49–51]. Furthermore, limited access to female condoms [40, 51] and a lack of information about condom use and its efficacy [40, 49, 51] were associated with inconsistent use of condoms.

### Sexual satisfaction and life after HIV

The majority of the Sudanese, Iranian, and Turkish WLHIV participating in the reviewed studies reported they had acquired HIV through sexual contact with their husband/partner with many expressing concern regarding their sexual life and satisfaction post-diagnosis [39, 41, 47, 49, 51]. Studies in Bangladesh, Iran, and Morocco suggested that most WLHIV remained married and sexually active [40, 44, 49]. The primary reasons for maintaining sexual activity was to stop their husband/partner from having extramarital sex [40], prevent conflict and harm [48], and to become pregnant [42, 49]. However, for some WLHIV, their HIV diagnosis had disrupted their sexual life and lowered their sexual pleasure [40, 44, 49].

A study in Morocco found that almost half of the 72 WLHIV chose sexual abstinence, nearly a quarter felt a loss of sexual desire, and there was an increased prevalence of sexual disorders (including low desire, painful intercourse, lack of pleasure and orgasm) among WLHIV from 7 to 69% pre- and post-HIV diagnosis [44]. WLHIV considered that painful and stressful intercourse was related to their antiretroviral therapy, experiences of sexual violence, denial of their HIV diagnosis by self and others [40, 44], and fear of becoming pregnant [49, 51]. Some WLHIV chose sexual abstinence due to fear of infecting their sero-discordant partner [44]. Others described how the religious taboos surrounding non-marital sexual activities also influenced their sexual satisfaction and choice to remain abstinent [44].

Studies in Iran and Kazakhstan [45, 48] described WLHIV’s dissatisfaction with their personal relationships. For some, their HIV diagnosis yielded loneliness, disappointment, and increased experiences of intimate partner violence. WLHIV from a number of studies described experiences of being sexually, physically, and emotionally abused by their partners along with restricted access to financial resources and support [43, 45]. This was particularly noted among women living in sero-discordant relationships [41, 48]. Many WLHIV who reported intimate partner violence stated they experienced a sense of helplessness in stopping or reporting this violent behaviour and could not leave the abusive relationship due to fear of stigma, social rejection, homelessness, and the future of their child [41, 48]. In the Kazakhstan study by Davis et al. [43], in which 23% of 242 respondents were commercial sex workers, recent sex trading was associated with intimate partner violence. Jiwatram-Negron et al. [45] indicated that women’s reporting sex trading was associated with the risk of gender-based violence, food insecurity, poor social support, mental illness, and drug use.

## Discussion

This review synthesized the existing literature to gain greater understanding of the complex social, cultural, and structural factors influencing the SRH needs and rights of WLHIV in Muslim-majority countries. It highlights the challenges WLHIV face in achieving their SRH rights, such as fertility desire, contraceptive use, perinatal care, and their optimal SRH. This review also draws attention to the complex array of cultural, religious, structural, and political barriers to achieving optimal SRH and well-being faced by WLHIV in Muslim-majority countries. Health and well-being are associated with cultural and social beliefs and norms, social class and religion [14, 52], contextual understanding of how these factors intersect with the broader determinants of health is key to WLHIV in Muslim-majority countries realizing and enjoying their SRH rights.

Fertility desire emerged as one of the important issues associated with WLHIV’s SRH rights [39–42, 46, 49, 51]. Consistent with previous studies exploring fertility intention among women in Muslim-majority countries [53–55], this review identified that social, cultural, and religious norms emphasizing the importance of having children in marriage and gender dominance within relationships directed the WLHIV’s fertility choices, often forcing women to actively seek motherhood regardless of their personal desire. Health care providers’ discriminatory attitudes, including actively suggesting abortions and discouraging WLHIV from having future pregnancies were commonly described in papers included in this review as factors influencing WLHIV fertility desire and outcomes. This result is consistent with other research conducted in Muslim-majority countries that found a strong moral view about HIV among health professionals created stigma and experiences of discrimination and affected how or if PLHIV accessed health care services [8, 56]. In contrast, recent evidence from studies in some non-Muslim majority countries in Asia and Africa with similar socio-cultural and gender-based normative beliefs demonstrate that WLHIV’s needs have in some cases started to be heard, respected, and supported by partners, families, and health care services and this has started to influence the incidence of unintended pregnancy and maternal-to-child transmission [57–59].

This review found that social and structural barriers, including lack of financial support, religious belief, stigmatizing and discriminatory attitudes of the health care providers, and limited understanding of and access to adequate contraceptive methods were associated with unmet contraception needs among WLHIV. Low prevalence of consistent condom uses or other modern contraceptives use was revealed in the reviewed studies [40, 49–51]. In line with previous studies in Africa [60–63], inconsistent condom use was linked to partner’s childbearing desire, women’s powerlessness to negotiate condom use, and women’s fear to disclose their HIV diagnosis [64–66]. Religious beliefs interfere with condom use in Muslim communities as many people who follow or practice Islam perceive that male circumcision and other religious customs protect them from acquiring HIV and other STIs and condom use is associated with immoral behaviours such as extramarital sexual activities which are not condoned under Islamic law [8, 67, 68]. Previous research highlights that many Muslims believe that contraception is allowed according to Islam, but use is discouraged [69, 70].

This review also found that experiences of stress during pregnancy, resulting from discriminatory treatment and inadequate information about mother-to-child transmission, impinged on the WLHIV’s fertility desire [41, 42, 49]. Further, echoing current research about WLHIV in other countries [71–75], this study suggests that an HIV diagnosis may increase the risk of women having adverse pregnancy outcomes. The consequences for the mother and baby are more complex and severe when WLHIV cannot access safe healthcare services and are discriminated against by healthcare providers [41, 42, 49].

The WLHIV in the reviewed studies commonly chose or were forced to become less sexually active post-HIV diagnosis, experienced changes in their level of sexual satisfaction, and reported numerous sexual disorders [40, 44, 48, 49, 51]. Similar to the findings of studies involving women from the predominantly Muslim population in Bangladesh, Iraq, Pakistan, and Indonesia [76–79], this review indicated that fear of divorce or forced separation and socio-economic dependence on their partner disempowered women and resulted in their inability to refuse coercive sex and leave or report abusive relationship [40, 41, 43–45, 48, 49]. Fear of transmitting HIV, becoming pregnancy and a loss of sexual desire were also identified as barriers to sexual satisfaction for WLHIV post-HIV diagnosis [44, 49, 51]. These findings are supported by the results of recent studies conducted in African, European, South American, and Asian countries [80, 81] that reported a lack of communication between WLHIV with their partner regarding safer and satisfying sexual practices led to sexual inactivity and dissatisfaction.

WLHIV in the reviewed studies were not always able to act on their sexual and reproductive choices, including fertility desire, condom use, and sexual interest, as they were not able to communicate their own needs and wanted to avoid conflict with their partner. Lack of protection of these WLHIV SRH rights raises concerns of repeated intimate partner violence [40, 45, 51], unplanned pregnancies [49, 51], sexual disorders [40, 44], STI, and mother-to-child transmission [42, 51]. Similar to other Muslim-majority country studies, this study found that social, political, and ideological conflicts hinder and threaten women’s SRH rights and well-being and fail to protect them from harmful behaviours [19, 23, 82, 83]. Cultural sensitivity may inhibit Muslim women from openly discussing reproductive, sexual, and HIV/STI issues [40, 84–86]. Religious belief associating HIV infection with immoral behaviour, such as sex outside marriage, intensifies stigma towards WLHIV and exposes women to discrimination, isolation, and in some cases harm [41, 46, 48].

The authors acknowledge that despite identifying a range of common SRH issues, the publications included in this review were limited in number and conducted in only a small number of the Muslim-majority countries. The findings and discussion presented may therefore not be generalizable to the broader Muslim-majority country context. Nonetheless, the results of this review highlight some key areas requiring urgent exploration and consideration when designing and implementing future SRH interventions and care for WLHIV and for all women in Muslim-majority countries. Religious, cultural, and political positions in predominantly Muslim populations hinder the comprehensive realization of women’s SRH rights, which is an urgent priority [19, 20].

## Conclusion

To conclude, the sexual and reproductive health needs and rights of WLHIV in Muslim-majority countries are frequently ignored due to socio-cultural, religious, political, and gendered norms and beliefs. Our findings acknowledge the importance of considering the SRH issues of WLHIV from their own perspectives and experiences, and to link these with contextualised understanding of the interrelated socio-religious-cultural and political factors influencing their fertility, contraceptive, and perinatal decisions and sexual life. Public awareness in Muslim-majority communities needs to be strengthened in reconstructing gender norms, socializing safer sex practices, reducing HIV-related stigma, protecting WLHIV from social, cultural, and religious violence, and empowering them to bravely voice their own SRH rights and needs. Further related studies and future SRH policy and interventions for WLHIV, particularly those in Muslim-majority countries, need to be informed and developed based on this understanding.

## Data Availability

All data generated or analysed during this study are included in this published article.

## Abbreviations

ART: Antiretroviral Therapy
HIV: Human Immunodeficiency Virus
MMAT: Mixed methods appraisal tools
SRH: Sexual and reproductive health
STI: Sexually transmitted infections
WLHIV: Women living with HIV
